# The Structural and Functional Organization of Cognition

**DOI:** 10.3389/fnhum.2016.00501

**Published:** 2016-10-17

**Authors:** Peter J. Snow

**Affiliations:** School of Medical Science, Griffith UniversityGold Coast, QLD, Australia

**Keywords:** prefrontal cortex, emotional intelligence, practical intelligence, temporal intelligence, abstract intelligence, metacognition, evolution of language, nature of lying

## Abstract

This article proposes that what have been historically and contemporarily defined as different domains of human cognition are served by one of four functionally- and structurally-distinct areas of the prefrontal cortex (PFC). Their contributions to human intelligence are as follows: (a) BA9, enables our emotional intelligence, engaging the psychosocial domain; (b) BA47, enables our practical intelligence, engaging the material domain; (c) BA46 (or BA46-9/46), enables our abstract intelligence, engaging the hypothetical domain; and (d) BA10, enables our temporal intelligence, engaging in planning within any of the other three domains. Given their unique contribution to human cognition, it is proposed that these areas be called the, social (BA9), material (BA47), abstract (BA46-9/46) and temporal (BA10) mind. The evidence that BA47 participates strongly in verbal and gestural communication suggests that language evolved primarily as a consequence of the extreme selective pressure for practicality; an observation supported by the functional connectivity between BA47 and orbital areas that negatively reinforce lying. It is further proposed that the abstract mind (BA46-9/46) is the primary seat of metacognition charged with creating adaptive behavioral strategies by generating higher-order concepts (hypotheses) from lower-order concepts originating from the other three domains of cognition.

## Introduction: Cognition and the Prefrontal Cortex

“The specific histological differentiation of the cortical areas proves irrefutably their specific functional differentiation…the large number of specially built structural regions points to a spatial separation of many functions and…the sharply delimited localization of the physiological processes which correspond to it.”

*Korbinian Brodmann (1868–1918) German Neurologist and Neuroanatomist.*

Understanding the nature of human intelligence is perhaps the greatest challenge to modern neuroscience, promising not only a comprehension of how humans have used cognition to dominate all other life forms, but also opening the possibility of replication of our mental faculties in the creation of systems that match or potentially exceed, our own extraordinary, cognitive abilities. Although intelligence is usually referred to in the singular, there is considerable evidence that it exists in multiple forms that potentially reflect different cognitive centers (Gardner, 1993; Gray and Thompson, 2004).

The existence of multiple intelligences is implicit in the studies of the development of cognition throughout childhood (Piaget et al., 1970, 1973). Thus, the development of cognition throughout childhood has been shown to be divided into the following stages: (i) the preoperational (1–6 years), when the child’s preoccupations are almost entirely in the psychosocial domain; (ii) the concrete operational (7–11 years), when the child fully engages the physical/factual domain; (iii) the formal operational (11–15), when the child engages successfully in the hypothetical, abstract domain, while, in addition, Piaget also regarded time as a discrete cognitive ability that enables the co-seriation of tasks (Piaget and Pomerans, 1969; Sauer, in preparation). Alongside the development of the child’s abilities to conceptualize the psychosocial, material and hypothetical realms, it is now widely recognized that the ability to plan or conceptualize the future is a fourth, emergent domain of human mentality that is responsible for what I will refer to as temporal intelligence (Piaget and Pomerans, 1969; Allen et al., 1991; Benson, 1997); a nomenclature for a cognitive domain that has very recently been recognized as a necessary component in the creation of robotic intelligence (Maniadakis and Trahanias, 2011, 2014, 2016; see: “The Origin of the Future: The Temporal Mind” Section).

Modern studies of cognition have confirmed previous suspicions that cognition is a function of specific parts of the prefrontal cortex (PFC) the activation of which is accompanied by the experience of thought (MacLean, 1990; Searle, 2000). From their pioneering studies of the monkey PFC, Levy and Goldman-Rakic have proposed “a modular “domain-specific” model of PFC’s functional organization with respect to working memory (WM) operations. In this model, the dorsolateral PFC (DLPFC) is composed of several subregions, based primarily on the nature of the information being processed in WM. Storage and processing functions are integrally related in each area (Levy and Goldman-Rakic, 2000, p. 23). Because all the cytoarchitectonic areas in the human PFC are present in macaque monkeys (Petrides and Pandya, 1999; Petrides et al., 2012) and probably the apes (Semendeferi et al., 2001) it would be reasonable to ask, whether, although greatly attenuated, monkeys and/or apes might be availed of cognitive domains more or less identical to those identified in humans?

It is well known that apes, but not monkeys, can perform Theory of Mind tasks (Byrne, 1995; Call, 2007; Call and Tomasello, 2008; Poulin-Dubois et al., 2009) and so qualify for having a modicum of emotional intelligence. Furthermore, apes and some monkey species have been observed making simple tools, testifying that both groups of primates can manifest practical intelligence (Byrne, 1995; Bortolini and Bicca-Marques, 2007; Lonsdorf et al., 2010; Haslam et al., 2013). Many studies of cognition in the chimpanzee and the closely-related Bonobo or pigmy chimp have shown significant domain-related differences indicating that Bonobos are dominated by emotional intelligence while practical intelligence exerts more control over chimp behavior (McGrew, 1992, 2004; de Waal, 2006; Waal, 2007; Matsuzawa, 2013). This hypothesis is supported by Herrmann et al. (2010; p. 1) who upon comparing the performance of chimpanzees and Bonobos on a wide range of cognitive problems designed to test their understanding of the physical and social world, concluding that; “Bonobos were more skilled at solving tasks related to Theory of Mind or an understanding of social causality, while chimpanzees were more skilled at tasks requiring the use of tools and an understanding of physical causality.” However, although Call has proposed that chimpanzees are “capable of knowledge abstraction to solve novel problems” (Call, 2001, p. 338), it is extremely difficult to design experiments to reveal abstract intelligence in non-human primates (Byrne, 1995; Milner and Goodale, 2008; Snow, 2009, 2014; MacLean, 2016; Rosati and Santos, 2016). Nevertheless, it is this domain of abstract intelligence that matures at the end of Piaget’s formal operations stage, that is both the defining feature of humanity and the origin of its innate ability to, in the practice of science, transcend the physical and sensibly address the hypothetical.

There are only two alternative hypotheses that explain how, during childhood the PFC could sequentially elaborate four qualitatively different cognitive operations to serve the psychosocial, material, temporal and hypothetical domains of human thought. The first, postulates that all four of these radically different domains of cognition are serviced by a single area of cortex. If this were the case, then between the beginning of the preoperational stage, at an age of approximately 1 year, until the establishment of the ability to perform formal operations, around the end of the 15th year, this single area of cortex would have to: (a) sequentially enable the development of each of the four, vastly different, operational systems (neural networks) required for emotional, temporal, practical and abstract intelligence; and (b) maintain the independence of these four domains of cognition, throughout adult life. Under this hypothesis, the neural network responsible for emotional intelligence would have to mature first, followed by the progressive maturation of the neural networks underlying temporal, practical and finally, abstract intelligence. Yet, each would have to retain its isolation and independence of action. If such an area of the cerebral cortex were to exist, then it would essentially constitute the human mind.

While this first proposal is, theoretically, possible, suffice it to say that the vast numbers of studies of cortical function have yet to elucidate any area of cortex which can simultaneously serve such radically different functions. Indeed, such a proposition would be attune to suggesting that the diverse sensations of vision, touch and hearing could each be effectively processed and meaningfully represented by a single cortical area. It follows that this first hypothesis is simply untenable.

The second hypothesis postulates that, the enabling of each domain of cognition is dependent on the progressive maturation of one of four circumscribed areas of the PFC. In this process each area slowly becomes operational on a time course paralleling the progressive appearance of the child’s cognitive abilities to conceptualize the psychosocial, material and hypothetical, while the temporal domain engages with each in the progressive development of the child’s ability to make plans. This proposal is completely compatible with the rules that govern the development of all cytoarchitectonic areas of the post-PFC, wherein: (a) each area of Brodmann serves a discrete sensory, perceptual, motor or mnemonic function and can be shown to have an identifiable place in human experience (Snow and Wilson, 1991; Snow, 2009, 2014); and (b) the PFC undergoes continuous structural and functional changes throughout childhood that are accompanied by the progressive emergence of all four cognitive domains (Casey et al., 1997, 2000; Diamond, 2000, 2002; Sowell et al., 2001; Adleman et al., 2002; Durston et al., 2006; Huizinga et al., 2006; Rosenberg-Lee et al., 2011; Baron and Leonberger, 2012; Dumontheil, 2014).

A vast amount of data have repeatedly implicated Brodmann’s areas (BAs) 9, 10, 46 or 47 in specific cognitive challenges, ranging from Theory of Mind tasks (Goel et al., 1995) to the Stroop test (Friese et al., 2013). By necessity, these cognitive challenges are always part of a specific protocol and a specific experimental paradigm designed to test which of these areas plays a role in that particular contemplative process. It is hoped that from the careful documentation of the participation or otherwise of each area in a multidomanial array of tasks, eventually the functional identity of each will emerge, enabling us to comprehend the constitution and variety of these cognitive centers that, collectively, constitute the human mind. The proposal that each cognitive area is the origin of a distinct domain of thought is particularly interesting with respect to: (a) the documentation of the large variation in the size of BAs 9 and 46 in a small sample of normal adult brains (Rajkowska and Goldman-Rakic, 1995); and (b) the malformation/function of these areas in schizophrenia (Selemon et al., 1995; Guillozet-Bongaarts et al., 2014) and autism (Critchley et al., 2000a; Schultz et al., 2000; Pierce et al., 2001).

**Figure 1:**
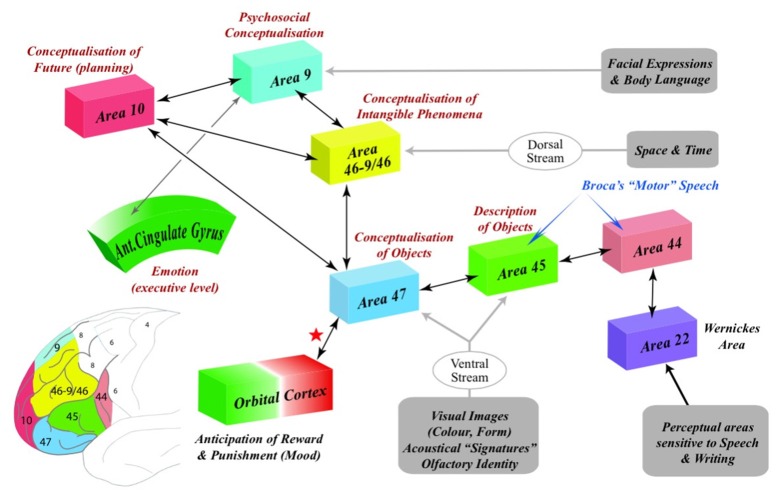
**The representation of the psychosocial, practical, temporal and abstract domains of cognition within Brodmann’s areas 9, 10, 47 and 46-9/46 (BAs 9, 10, 46-9/46 and 47).** The social mind (BA9) receives information from areas of the cortex that enables us to integrate facial expressions, gaze and body language of other agents with our own emotions. The ventral stream delivers information to the material mind (BA47) and the anterior part of Broca’s area (BA45) from cortical areas that enable us to perceive the identity of objects and people. In contrast, the dorsal stream delivers information about the space and time to the abstract mind (BA46-9/46) that is not perceptual in nature (Milner and Goodale, 2008, p. 775). In order to accommodate this perceptually-barren information from the intangible phenomena of space and time, it is proposed that the circuitry within BA46-BA9/46 reorganizes enabling it to embody the necessarily incongruent inputs from the social (BA9), material (BA47) and temporal (BA10) domains of cognition into hypothetical concepts that characterize Piaget’s Formal Operations stage of cognitive development (see: Figure 2). The temporal mind (BA10) engages the psychosocial, practical or abstract domains of cognition in the specific cognitive processes involved in planning. Note that both Broca’s area and BA47 participate in both gestural and verbal communication indicating that the evolution of language is intimately linked to practicality. Note also the interaction (red star) of the material mind (BA47) with the orbital cortex (BA11) that underlies the autonomic arousal (negative reinforcement) which results, specifically, from lying about concrete facts.

The purpose of this article is to propose specific cognitive functions for BAs 9, 10, 46 and 47 that are implicit in the great majority of studies and that match a long history of pre-existing ideas on the nature of human thought (Figure 1).

## The Origin of Emotional Intelligence: The Social Mind

Complex social interaction is a hallmark of the human species. More than any other arena of life, our social interactions are intimately linked to our emotions, and, as such, inscribe themselves, almost indelibly, into our episodic and emotional memories (Kensinger, 2009). Yet many of the vehicles of social interaction, such as the reading of body language and mimicry, can be identified in the apes and doubtless characterized the behavior of the australopithecines and our hominid ancestors (Snow and Wilson, 1991; Byrne, 1995; Call, 2007; Call and Tomasello, 2008; Poulin-Dubois et al., 2009; Snow, 2014). In non-human primates, there is an inherent reliance on social bonding, social ostracization often having catastrophic consequences (Byrne, 1995). In humans, socially-compromising, psychiatric conditions such as autism spectrum disorder (ASD) provide a poignant reminder of our dependence upon accurately interpreting and integrating the emotional states of self and others—an otherwise innately acquired ability that underwrites all social interactions (Frith, 2003; Blakemore et al., 2004).

### Emotional Intelligence and Interpersonal Communication

Our acute psychological dependence upon social interaction has led some to consider that all higher brain functions, including our capacity for abstract reasoning and metacognition, have evolved solely to service our need for society (Dunbar, 1993; Gobbini et al., 2007; Frith, 2012; Shea et al., 2014). Yet, in Piaget’s identification of the sequential development of very different cognitive stages throughout childhood, the creation of the fundamental, psychosocial concepts that are the foundations of emotional intelligence, are confined to early years (Frith and Wolpert, 2004). It is not until after this preoperational stage that the remainder of childhood sees the development of forms of intelligence which are not intrinsically concerned with social interaction but, instead, are almost exclusively focused upon the material, abstract or temporal domains of human contemplation (Piaget and Pomerans, 1969; Piaget et al., 1970, 1973; Snow, 2003, 2009, 2014; see: “The Origin of Hypotheses: The Abstract Mind” and “The Origin of Practicality: The Material Mind” Sections).

Individuals with ASD have difficulty in reading and expressing body language and, in severe cases, verbal language, manifesting in these ways a profound deficit in their comprehension of others (Frith, 2003). Yet people with ASD occasionally demonstrate astounding mental abilities in both practical and abstract domains (Frith, 2003; Mottron et al., 2013; Treffert, 2014). At the opposite extreme of the cognitive spectrum are individuals with Williams’ syndrome, whose behavior and speech are dominated by psychosocial concerns alongside a low intellectual ability and inherent weaknesses in visuospatial construction and tool use; deficits that are thought to reflect abnormalities in the ventral and dorsal streams (Morris, 2010; O’Hearn et al., 2011) that, as argued elsewhere in this article, are the defining inputs to BA47 (the material mind) and BA46 (the abstract mind), respectively. While in day-to-day life the cognitive stance of a normal person might most easily be interpreted as “social”, the hypersocialibilty of individuals with Williams syndrome does not overcome their asocial cognitive disabilities, while the socially-barren, cognitive extremes seen in ASD, powerfully contests the idea that emotional intelligence is either consistently dominant or necessary for survival in the modern world. Indeed, it has been suggested that ASD represents an extreme of a continuum that describes the normal male psyche (Baron-Cohen, 2002; Hall et al., 2012).

### Localizing Emotional Intelligence—Early Studies

In the search for the source of emotional intelligence, early brain imaging studies soon focused on BA9—an area that composes the medial PFC (mPFC)—each of these terms being used by different authors. Activation of BA9 was observed in response to a host of personal or people-related stimuli, activities or scenarios, such as tickling, pain, swallowing, sensing familiar odors, rhyming words, reporting on the pleasantness vs. unpleasantness of words (McDermott et al., 1999), judging the recency of conversation (Zorrilla et al., 1996), the retrieval of autobiographical episodic memories (Lepage et al., 2000) and the implementation of action (MacDonald et al., 2000), hearing stories about interpersonal relationships, recalling or seeing film clips that portray the experiences of happiness, sadness or disgust (Fletcher et al., 1995; Lane et al., 1997a; Shallice, 2001; MacLean, 2016), seeing pictures of unpleasant themes such as frightened animals, mutilated bodies or human violence, or pictures of pleasant themes such as erotica, babies or sports events (Lane et al., 1997b) and performing Theory of Mind tasks (Goel et al., 1995). Conversely, emotionally neutral pictures showing inanimate objects, people with neutral facial expressions or complex scenes or patterns failed to activate BA9 (Lane et al., 1997b). Finally, there was abnormally low activation of BA9 in subjects with autism or Asperger’s syndrome (Happe et al., 1996; Ohnishi et al., 2000). The involvement of BA9 in emotional intelligence is also indicated by the findings that it is histologically abnormal in schizophrenics (Selemon et al., 1995; Guillozet-Bongaarts et al., 2014)—a condition that compromises a person’s ability to comprehend the intentions of others (Frith and Frith, 1999). When brain imaging of schizophrenics was performed during Theory of Mind tests, emotion processing and the judging of agency, there was, as in autism and Asperger’s syndrome, a reduced recruitment of BA9 (Brunet-Gouet and Decety, 2006; Dodell-Feder et al., 2014).

### Emotional Intelligence and the Social Brain

Psychologists and more recently neuroscientists have postulated the existence of the *social brain*; a network of interconnected neural centers devoted to maintaining our community with our fellow beings (Dunbar, 1993; Frith, 2007; Blakemore, 2008). Consequently, modern brain imaging studies have focused on identifying functional networks of specific areas that are activated by psychosocial challenge and therefore qualify as part of the social brain. These studies have revealed functional connections (as distinct from anatomical connections) between specific areas in both the prefrontal and post-prefrontal areas of the cortex—areas that are coactivated in socially relevant tasks ranging from simply identifying facial expressions to transiently representing the intentions of others (Van Overwalle, 2009, 2011; Koban and Pourtois, 2014; Li et al., 2014). When, however, the prefrontal lobes are excised from the mature brain, only the post-prefrontal elements of the social brain remain, areas that presumably enable the survival of behavioral phenomena such as mimicry, face recognition and coordinated movement, alongside the virtual annihilation of the ability to conceptualize and reason—an observation emphasizing that the neural circuitry underlying all forms of significant mental activity is located within the PFC (MacLean, 1990). With respect to emotional intelligence, Van Overwalle (2009) concludes that when a task requires the preservation in WM of more enduring elements of human interaction, such as the emotional disposition of self and others, there is a consistent participation of the mPFC. It follows that the social brain must constitute a network of post-prefrontal centers that pass on highly integrated information to a unique, functional and structural entity within the PFC that supports the algorithms of emotional intelligence (Gardner, 1993; Goleman, 1995, 2007).

With respect to the concept of a social brain, brain imaging has shown that a large number of prefrontal and post-prefrontal areas may participate in social interactions. Post-prefrontal sites include superior temporal sulcus (STS, biodynamic imagery), temporo-parietal junction (TPJ, inferring goals of others), inferior parietal lobule (IPL, inferring motor intentions of others), posterior cingulate cortex (PCC, explicit and implicit emotional engagement), anterior insula (AI, receives visceral afferents, emotional awareness), fusiform face area (FFA, sense of agency), orbital frontal cortex (OFC, prediction of reward or punishment) and the amygdala (elaboration of fear or aggression; Van Overwalle, 2009; Gu et al., 2013; Koban and Pourtois, 2014; Li et al., 2014). Prefrontal sites include the anterior cingulate cortex (ACC; BAs 24, 32, 33, representation of emotional states), mPFC (or BA9, enduring psychosocial concepts) and the inferior frontal gyrus (IFG or BA47, referred to by some authors as the ventrolateral PFC [VLPFC]; see: “The Origin of Practicality: The Material Mind” Section). Given its role in practicality, the recruitment of BA47 in the meeting of particular psychosocial challenges is at first puzzling. However, examination of the specific experimental paradigms used in these cases always reveals a requirement for concrete or physical identification. For example, when subjects viewed pictures of human suffering the mPFC (BA9) was activated, while viewing pictures of dog suffering activated the IFG (BA47), indicating that, in the latter case, subjects made a practical, objective, semantic evaluation rather than one involving psychosocial issues (Franklin et al., 2013). Similarly, when males and females make social decisions from faces, the IFG participates more strongly in males, an effect that is even more exaggerated in ASD wherein the utility and functional identity of others far outweighs their emotional significance or their social identity (Hall et al., 2012). On these arguments and those made elsewhere in this article (see: “The Origin of Practicality: The Material Mind” Section), caution should be taken before including the IFG (BA47) in propositions regarding a social brain network.

### Differentiating Cognition from Perception in the Social Brain

In his detailed meta-analysis of social cognition, Van Overwalle (2009) states that “…the conclusion that enduring trait and norm inferences crucially involve the mPFC seems overwhelmingly supported by the empirical data, whereas the TPJ seems additionally engaged for processing visual material about others. This provides support for the view that the understanding of humans as enduring organisms with permanent social and psychological properties such as traits and norms is a crucial common element that engages the mPFC” (Van Overwalle, 2009, pp. 847–848). Unfortunately, for the present case, Van Overwalle chose to consider the ACC and BA9 as a single functional entity for which he uses the term mPFC. It is therefore not possible to use his data to better localize psychosocial cognition to either of these two prefrontal areas. He does, however, comment that, “Other general-purpose functions engage areas of the PFC that are not activated during social cognition, and are therefore very unlikely to constitute a core process of social inference, contrary to ideas put forward by many authors in the social neuroscience literature… and social psychology…” (Van Overwalle, 2009; p. 850), thus making an important point regarding the localization of different domains of cognition in other parts of the PFC (Barrett et al., 2007).

In a recent review, Koban and Pourtois (2014, p. 5) state that “…evidence indicating that dorsal ACC, or, more broadly, the dorsal mediofrontal cortex (dMFC), is involved in both cognitive and emotional processing…and could therefore constitute an important hub for emotion–cognition interactions”. Again, however, this statement does not differentiate between the ACC and BA9 with respect to a role in cognition. Most sub-primate mammals have a relatively minute PFC composed largely of cortex that is functionally homologous to the orbital and cingulate cortex of primates (Uylings and van Eden, 1990; Snow et al., 1992; Uylings et al., 2003; Bush and Allman, 2004; Snow, 2009, 2014; Van De Werd et al., 2010; Kaas, 2013). With little if any cortex to spare for cognition, the behavior of non-primate mammals must be wholly dependent upon an integration of instinctual drives (raw emotions) with memory traces (emotional memories) archived in the ACC, amygdala and the orbital cortex (MacLean, 1990; Snow, 2009, 2014). There can be little doubt that the absence of appreciable amounts of PFC accounts for why it has been so difficult to prove the existence of cognitively organized, strategically-based behavior in non-primate mammals (Byrne, 1995; Mulcahy and Call, 2006; Suddendorf, 2006; Suddendorf and Corballis, 2007; Osvath, 2009; Merritt et al., 2010; MacLean, 2016). The apparent sophistication of the ACC in humans, most likely reflects our pre-primate origins when, in the absence of significant amounts of cortex devoted to cognition, the ACC would have constituted the highest, “executive” level of the central nervous system (MacLean, 1990). However, rather than a cognitive center in its own right, it is more likely that the ACC of the human brain acts as an, albeit sophisticated, executive station for the representation of distinct classes of emotions rather than participating directly in the processes of thought (Snow, 2009, 2014). Indeed, the neurological report of a patient who recovered from a state of akinetic mutism precipitated by a temporary loss of function in her ACC did not indicate a loss of cognitive ability but, instead, that she comprehended (was cognitively aware of) what was said to her but suffered a total loss of motivation required to take action (Damasio and van Hoesen, 1983).

In their review of functional networks involved in social understanding, Li et al. (2014) consider the ACC and mPFC as separate functional entities, such that their term, mPFC, can only be spatially and functionally synonymous with BA9. Thus, with respect to the function of BA9, they conclude that, “Common results show that the MPFC plays a key role in the social understanding of others, and the subregions of the MPFC contribute differently to this function…[thus] the ventral MPFC…and its connections with emotion regions are mainly associated with emotion engagement during social interactions…[while]…the anterior MPFC (aMPFC)…and its connections with posterior and ACC contribute mostly to making self-other distinctions…[and]…the dorsal MPFC (dMPFC)…and its connection with the TPJ are primarily related to the understanding of other’s mental states” (Li et al., 2014, p. 1). As these conclusions are not related to ACC but specifically to BA9, it seems reasonable to conclude that there is a functional subdivision of labor within BA9 that embraces three different subcategories of psychosocial cognition. Restated in terms of BA9, these are: (a) the dorsal part of BA9 (dBA9) supports an understanding of the mental states of others or Theory of Mind processes; (b) the anterior part of BA9 (aBA9) interacts with the ACC and the PCC in making self-vs.-other distinctions; and (c) the ventral part of BA9 (vBA9) participates in emotional engagement during social interactions or, more specifically, controlled, cognitively-evoked empathy (Snow, 2009, 2014). While it is clear that the processes of psychosocial cognition must draw upon an ensemble of complex perceptions, such as the emotional status of the host and the emotional readings of other agents, the analysis of Li et al. (2014) provides powerful evidence that the cognitive element within our social brain’s network is BA9 an area that supports “reasoning about the thoughts and intentions of conspecifics” (MacLean, 2016, p. 6917).

I therefore propose that, as BA9 constitutes a unique, anatomically-distinct element of human cognition that engages the psychosocial domain of life, it be known as the social mind, an organ of the brain that is responsible for our emotional intelligence (Figure 1).

## The Origin of Practicality: The Material Mind

Our virtually unique ability to name, categorize and classify thousands of objects and to use them to construct a vast array of new objects has led some to suggest that, rather than *Homo sapiens*, man the knower, a more apt name for our species might be *Homo faber* or man the maker (Huizinga, 1970). Until recently, the ability to fashion simple, oldowan tools was believed to have first appeared in *Homo habilis* (man the able), which is also the first hominid generally considered to be capable of verbal communication (Leakey et al., 1964). However, it has been shown that the metacarpals of the hand of *Australopithecus africanus* enable the forceful opposition of the thumb and fingers which is considered important for the construction of tools (Skinner et al., 2015) and in accord with this, crude stone tools have been discovered that were constructed 3.3 million years ago or 700,000–800,000 years before the oldest remains of *H. habilis* (Harmand et al., 2015).

### Practical Intelligence and The Evolution of Language

The evolution of language is often considered to be a consequence of our dependence on a complex social structure (Valsiner and Veer, 2000; Cheney and Seyfarth, 2007; Lee, 2009). However, without speech, apes use body language, gesturing, facial expressions and relatively crude vocalizations to successfully maintain a complex social organization (Lieberman, 1986; Byrne, 1995; McGrew, 2004; de Waal, 2006; Pollick and de Waal, 2007; Waal, 2007). As it is also well established that the majority of interpersonal communication between humans is dependent on body language and facial expressions (Pinker, 1994), it again seems reasonable to conclude that the selective advantage offered by verbal communication is related in only a minor way to the dependence of *Homo sapiens* or our hominid or pre-hominid relatives, on a complex social structure. Instead, it is more likely that language played a significant role in the transmission of knowledge that underpinned the ever-advancing stone tool industry, that is considered to reflect the progressive intellectual ascendency of the our hominid ancestors.

There is now considerable evidence that the evolution of verbal communication was brought about by the need of our hominid ancestors to accurately convey information about practical matters (King et al., 2011). Neuroscientific evidence that communication and practicality have a common phylogenetic origin is apparent from studies in the macaque monkey, which have shown that the prelinguistic precursor of Broca’s area (BA45/44) participates in both gesturing and manual tasks that are unrelated to communication (Greenfield, 1991; Arbib, 2005; Petrides, 2006; Petrides and Pandya, 2009; Yeterian et al., 2012). In humans, Broca’s area (BA 45/44) participates in the integration of speech and gesturing (Skipper et al., 2007; Gentilucci and Dalla Volta, 2008; Flinker et al., 2015) while both Broca’s area and its contralateral homolog are involved in hand-object interactions including tool usage (Schendan and Stern, 2007; Higuchi et al., 2009; Stout and Chaminade, 2012). Despite the imitative capacity of apes and humans (Byrne, 1995; Frith and Wolpert, 2004), the ability to accurately communicate factual knowledge and to physically manipulate objects together have a far greater potential for survival than either attribute operating in the absence of the other—again emphasizing the relative independence of language from selective pressures relating to the maintenance of a complex social structure in our hominid ancestors.

### The Integration of Practicality and Language

In each hemisphere, BA45 and BA44, together with the immediately-adjacent, anteriorly-placed BA47, compose the IFG. As a consequence of its proximity to BA45, many reviews of neuroimaging studies of language have implicated BA47 of the left hemisphere in the production of speech (Bookheimer, 2002; Schmidt and Seger, 2009; Bohrn et al., 2012; Rapp et al., 2012). Numerous other neuroimaging studies have, however, focused on the role of the left BA47 in the comprehension of objects which is a necessary prerequisite for their implementation in the manufacture of tools and all forms of construction (Ungerleider et al., 1998; Royet et al., 1999; Adams and Janata, 2002; Plailly et al., 2007; Renier et al., 2009; Garcin et al., 2012; Kjelvik et al., 2012). It follows that, if language and practicality share a common phylogenetic origin, then it may well be that the expansion of BA47 was the critical process in hominid evolution that united these vital elements of human aptitude.

Given the contiguity of Broca’s Area and BA47, it is not surprising that many studies of the IFG have focused on its potential role in the mechanism of language, thus limiting the possibility of establishing a potentially more fundamental role for this area. In a recent meta-analysis, Rapp et al. (2012) concluded that the anterior part of the left IFG (BA47/45) engages in the semantic processing or, more specifically, the integration of words into meaningful sentences (Bookheimer, 2002; Badre and Wagner, 2007; Menenti et al., 2009; Friederici, 2012; Rapp et al., 2012). In a second meta-analysis, Bohrn et al. (2012) report that when subjects attempt to comprehend non-literal, figurative language, nearly all studies report activation of the left IFG. When, however, subjects attempt to comprehend metaphors, the left IFG is activated in response to conventional metaphors, while the right IFG only participates in deciphering unconventional or novel metaphors (Bohrn et al., 2012).

In a study that specifically examined metaphor processing, Schmidt and Seger (2009) reported activation of the left anterior IFG (left BA47) when subjects encountered familiar metaphors and activation of the right anterior IFG (right BA47) in response to unfamiliar metaphors (Schmidt and Seger, 2009). To explain this asymmetry the authors call upon the “broader concept of salience”, hypothesizing that the right hemisphere is required for integration of words with less salient (less obvious) meanings (Giora, 1997, 1999, 2003; Schmidt et al., 2007).

In relation to this issue, it is important to appreciate that, in the context of our verbal language, salience (the most noticeable or important) is intimately bound to semantics (meaning in language or logic). On the other hand, in the context of the prelinguistic gesturing of our primate relatives (both apes and monkeys), salience is intimately bound to semiotics, the study of signs and symbols and their uses, meanings and interpretations (Stevenson and Oxford University, 2010)—a relationship that again highlights the inherent link between communication and our ability to conceptualize form and function that enables us to so effectively meet the practical challenges ever present within the material domain of human existence.

Rather than salience, however, success in naming objects appears to be determined more by a subject’s mental focus upon an object and its position in space (Einhäuser et al., 2008). Activation of BA47 in humans might therefore be indicative of its capacity to direct our attention to specific objects in accordance with their mentally conceived usefulness in either construction or communication (Roland and Gulyás, 1995; Facchini, 2000; Sakai et al., 2002; Curtis and D’Esposito, 2003; Schendan and Stern, 2007). In terms of the maturation of BA47 and the relationship between language and our practical aptitudes, it is relevant that the ability to comprehend and manipulate codes and anagrams while simultaneously becoming cognizant of the properties (parametric and symbolic) of objects and the rules that govern their use develops between the ages of 7–11 years, a period that constitutes Piaget’s concrete operational stage of cognitive development (Piaget and Pomerans, 1969; Piaget et al., 1970, 1973).

### Practicality and The Analysis of The Material World

From neurophysiological and early neuroimaging studies, Ungerleider et al. (1998; p. 882) concluded that “object WM in humans and monkeys are similarly located in VL PFC [IFG]”. The physical details of objects and their relative positions are conveyed to BAs 47, 45 and 44 via the ventral stream (Petrides and Pandya, 2002, 1999)—the “what” pathway of Goodale et al. (1991) and Goodale and Milner (1992). The IFG has also been implicated in the maintenance of information relating to objects (Courtney et al., 1998; Munk et al., 2002; Sala and Courtney, 2007), including their color (Elliott and Dolan, 1998; Mohr et al., 2003, 2006; Yee et al., 2003) and sound (Rama et al., 2004). Moreover, much neuroimaging work has shown that objects identified as familiar on the basis of vision, touch and hearing activate the IFG (Ungerleider et al., 1998; Adams and Janata, 2002; Renier et al., 2009) and are functionally categorized within the left BA47 (Garcin et al., 2012). Indeed, the IFG also participates in the olfactory identification of objects (Plailly et al., 2007; Kjelvik et al., 2012) with BA47 of the left hemisphere showing activation to the odor of familiar objects (Kjelvik et al., 2012).

Activation of BA47 and BA45 accompanies subjects making decisions about how to categorize objects. However, if they are asked to mentally rotate objects there is activation of both BA47 and BA46 but not BA45 (Roland and Gulyás, 1995; Schendan and Stern, 2007). In a departure from language-orientated protocols and the issue of familiarity, Garcin et al. (2012) have recently tested the involvement of the IFG in assessing the similarity of objects, stating that, “the neural basis of similarity detection [of objects] has never been studied because the hypothesis that specific areas are involved in such processing has not yet been formulated” (Garcin et al., 2012, p. 7). In their study, activation of the anterior IFG was measured while subjects were asked to associate one of the two drawings of objects with a drawing of a target object. In each trial, two forms of association were possible, one requiring the subject to seek similarity or difference in relation to a concrete, physical element, such as shape and the other requiring subjects to seek, what Garcin et al. (2012) refer to as similarity or difference in relation to category—this latter task requiring the divination of a thematic or functional link between two objects.

Garcin et al. (2012) report that while the anterior IFG in both left and right hemispheres was activated by comparisons of shape only the left anterior IFG was activated by comparisons of functional or thematic categories. These authors also cite neurological video evidence that patients with prefrontal damage have difficulty in comprehending similarities between objects in categorization tasks. It is important to note that Garcin et al. (2012) emphasize that, when their subjects were mentally focused on objects, it was the anterior part of both left and right IFG that was activated, which, in cytoarchitectonic terms, corresponds to BA47 and not Broca’s area (BA45/44) or its contralateral homolog. Moreover, in relation to the role of BA47 in contemplation of the material world, Garcin et al. (2012) conclude that the participation of the anterior IFG in “similarity detection may depend on more elementary sub-processes such as maintaining the intention to search for identity, building mental representations of abstract and/or concrete features for all items” (Garcin et al., 2012, p. 7).

### Practicality in Construction and Symbolism

In their review, Courtney et al. (1998) found that activation of the left IFG reflects analytical processing of visually presented objects, while activation of the right IFG is involved in processing image-based, iconic features of faces and objects. Since then, Vignal et al. (2000) have shown that electrical stimulation of the right IFG caused subjects to experience a rapid succession of faces (Vignal et al., 2000), while Downar et al. (2002) have demonstrated activation of BA47 of the right hemisphere occurs in accord with the novelty value of objects (Downar et al., 2002). These results approximate those of Garcin et al. (2012) in that they implicate the left IFG in the comprehension of the parametric properties of objects that are essential in defining both their similarity and their usefulness, while the right IFG attends to the shape of objects in relation to their iconographic or symbolic significance.

With respect to the interpretation of language, these studies accord not only with the classical dichotomy of hemispheric function (Springer and Deutsch, 1997) but also with the finding that BA47 in the right hemisphere is selectively employed in the complex semantic task of deciphering unconventional or unfamiliar metaphors (Schmidt and Seger, 2009; Bohrn et al., 2012)—metaphors that are often used when we attempt to verbally describe the symbolic and iconographic significance of certain classes of objects or unfamiliar, yet clearly symbolic, characters.

### Rules, Syllogisms and Practicality

A number of neuroimaging studies have implicated the IFG in the maintenance of task rules (Bunge et al., 2003; Sakai and Passingham, 2003, 2006; Bunge, 2004; Rowe et al., 2007; Bengtsson et al., 2009; Bode and Haynes, 2009). More recently, Reverberi et al. (2012c) have shown that, when subjects were asked to retrieve, maintain and apply sets of conditional rules to different target stimuli, BA47 in the right PFC participated in identifying the rule (Reverberi et al., 2012c). In a second study where subjects were asked to remember and apply either simple or compound rules, BA47 of the right hemisphere was implicated in both the representation of compound rules and the decoding of them from information extracted from single rules (Reverberi et al., 2012b). In contrast, BA47 in the left PFC has been shown to participate in the search for logically valid solutions to problems, the formal structure of which is represented within Broca’s area (BA45/44; Reverberi et al., 2012a).

Reverberi et al. (2012a) conclude that BA47 is involved in the “…selection and application of inferential rules” (Reverberi et al., 2012a, p. 1752), asserting, however, that its participation in the underlying deductive reasoning is dependent upon its interactions with other brain areas, specifically Broca’s area, the premotor cortex (BA6), the frontal eye fields (BA8) and the tertiary visual association cortex (V3 or BA19). While co-activation of neural centers is expected in behaving human subjects, the location of BA47 squarely within the networks of the PFC (Kelly et al., 2010; Thiebaut de Schotten et al., 2012; Yeterian et al., 2012) makes it reasonable to conclude that BA47 is the cognitive center of syllogistic deduction and is thus fundamental to the creation of rules applicable to our physical surroundings and their formulation into language.

### Practicality and the Definition of Lying

In the accurate communication of our physical reality, both observation and the selection of correct words are essential. Indeed in many circumstances that relate to our material surroundings miscommunication, errant observation or lying can be fatal. In contrast, the outcome of the truthful communication of either our psychosocial issues or our hypothetical beliefs is far less predictable and, in most cases, has so little bearing on survival that, for the sake of tact alone, we often intentionally avoid honesty in these particular cognitive domains.

Although, in the modern world, much factual communication is of little relevance to our survival, this would not have been the case for our socially-enabled hominid ancestors, who were seriously challenged with surviving the intense selective pressure of direct exposure to an often hostile environment. Under such conditions, any neural systems that would ensure the validity of factual communication would be of great selective advantage. Particularly in our hominid relatives, neither the communication of the hypothetical nor their prevailing moods would have matched the importance of the accurate conveyance of practical information about their environment or the participation of their cohorts in practical tasks.

The involvement of BA47 in so many matters that require accurate definition and logic suggests that, more than any other area of the brain, BA47 requires constraints that ensure the validity of communication. This is supported by the finding that BA47 is powerfully implicated in the definition of truth and validity. For example, neuroimaging studies of subjects telling spontaneous, isolated lies produced right hemisphere activation in both BA47 and BA11 of the lateral orbital cortex (Spence et al., 2004), an area known to be involved in the prediction of negative outcomes (punishment; Snow, 2003, 2009, 2014; Ressler, 2010; Sescousse et al., 2010). When, however, subjects consider their response to a question that allowed deception, the right BA47 was activated without activation of BA11 (Sip et al., 2012). In yet another paradigm, when subjects make false, compared to true, claims, the right IFG and amygdala were activated (Sip et al., 2013).

Anatomically, BA47 is dorsolaterally adjacent to, and interconnected with, BA11 (Kelly et al., 2010; Thiebaut de Schotten et al., 2012; Yeterian et al., 2012). In their study of logical reasoning, Reverberi et al. (2012a) have commented that BA47 is distinct from all other brain regions in its adherence to the principle of validity. In risk-taking behavior which lacks the assurance of either physical reality or syllogistic deduction, BA47 and BA11 of the right hemisphere are activated (Critchley et al., 2000b). The co-activation of BA11 with BA47 therefore represents a critical constraint experienced as a centrally-created negative reinforcement of lying that ensures sympathetic arousal specifically when concrete truths seated in the physical reality of our immediate environment are contravened (Snow, 2003, 2009, 2014).

This phylogenetic perspective and the role of BA47 in semantic processing, similarity detection, salience, semiotics, gesturing, familiarity, the comprehension of metaphors, the selection of inferential rules, syllogistic deductions and the identification of objects and the representation of their parametric and iconographic functionality, indicates that BA47 acts as a mental platform responsible for the elaboration and communication of human practicality. I propose, therefore, that, as BA47 constitutes a unique, anatomically-distinct element of human cognition, it be known as the material mind, an organ of the brain that is responsible for our high degree of practical intelligence (Figure 1).

## The Origin of the Future: The Temporal Mind

The loss of both the ability to plan and the ability to conceptualize the future are conspicuous consequences of prefrontal lobotomy (MacLean, 1990). More than any other single factor, planning involves a registration of the passage of time, for in many real life circumstances, time is the essence of survival (Piaget and Pomerans, 1969; Allen et al., 1991; Benson, 1997). In their review of time models and cognitive processes in robotic behavior, Maniadakis and Trahanias (2014) emphasize the need for a component that enables “the multimodal interaction between sense of time and cognition that goes far beyond the perception of duration, addressing a very broad range of cognitive functions. This suggests temporal cognition as the cognitive glue that enables the integration of skills into a coherent smoothly functioning composite system” (Maniadakis and Trahanias, 2014, p. 4).

### The Nature of Planning

When challenged with a complex goal, our cognitive centers automatically divide the task into a temporally-ordered series of smaller tasks or sub-goals that must be performed in the correct sequence in order to attain success. Plans differ from other elements of cognition in that they are never of the moment, but instead can only be actualized in the future and so must be preserved in memory. Planning is the basis of proactivity and as such, it demands the integration of time, both past (reflective) and future (prospective). It follows, that in order to orchestrate the steps of any plan, a neural center must be able to preserve the plan in what is almost universally referred to as prospective memory (Okuda et al., 1998; Koechlin et al., 1999, 2000; Burgess et al., 2001). If we consider that many human plans extend over days, weeks and months, then our ability to plan is indicative of a neural platform that enables us to meaningfully conceptualize the future—a process that inevitably draws upon our (episodic) memories of those past events that are germane to the success or failure of our current goals. Notwithstanding the retrospective nature of memory, the essential steps of planning are firmly directed towards the future and the necessity to remember a plan has therefore been referred to as a “memory of the future” (Ingvar, 1985). It is this “memory of the future” that is lost when the prefrontal lobes are disconnected from the rest of the brain (MacLean, 1990) or even when damage is confined to BA10 on the frontopolar surface of the prefrontal lobes (Roca et al., 2011).

### Localization and Activation of Temporal Intelligence

It has been widely recognized that while patients with lesions of the most rostral part of the PFC (BA10) remain cognizant of the contextual information that they would need to plan a sequence of actions, they consistently fail to appreciate the importance of temporally organizing their own actions in order to achieve any given objective (Grafman et al., 1993; Sirigu et al., 1995; Loarer et al., 1998; Swain et al., 1998; Burgess, 2000; Roca et al., 2011; Volle et al., 2011). The specific source of these deficits became clear when early neuroimaging studies implicated BA10 in the organization and registration of each sub-goal of a task (Baker et al., 1996; Owen et al., 1996; Koechlin et al., 1999, 2000; Braver and Bongiolatti, 2002) and the retention of both the status of each sub-goal and the overall plan in prospective memory (Pochon et al., 2002). Thus, Koechlin et al. (1999, p. 148) have stated, “bilateral regions in the frontopolar PFC (FPPC or BA10) alone are selectively activated when subjects have to keep in mind a main goal while performing concurrent (sub) goals. Neither keeping in mind a goal over time (WM) nor successively allocating attentional resources between alternative goals (dual-task performance) could by themselves activate these regions.”

When experimental designs have specifically targeted the elements required for planning, BA10 is repeatedly found to play a complex yet critical role in this essential human activity. Thus the rostral PFC (rPFC or frontopolar cortex, FPC) has been implicated in thinking about the future (Okuda et al., 2003), multitasking, episodic memory retrieval (Gilbert et al., 2006), thinking about intentions (den Ouden et al., 2005), relational reasoning, characterized by the need to consider many relations simultaneously (Christoff et al., 2001, 2003; Kroger et al., 2002; Tsujimoto et al., 2011), the simultaneous pursuance of two independent goals (Charron and Koechlin, 2010), encoding goals (Genovesio et al., 2012), lower-order rule switching (Yoshida et al., 2010), switching tasks (Dumontheil et al., 2010a), keeping “what” and “when” intentions in mind (Momennejad and Haynes, 2012), prospective visual-spatial memory (Costa et al., 2013), sustaining prospective, but not working, memory (Okuda et al., 2007; Reynolds and O’Reilly, 2009; Burgess et al., 2011), control of cognitive branching in the allocation of attention between two tasks (Koechlin et al., 2000; Koechlin and Hyafil, 2007), multitasking resulting from prospective memory of deferred goals (Koechlin et al., 1999; Burgess, 2000; Dreher et al., 2008; Burgess et al., 2009), switching of behavior in the exploration and exploitation of prevailing circumstances (Boorman et al., 2009), establishing task sets (Sakai, 2008), attentional sets (Pollmann, 2004) and cognitive, context-dependent sets (Rowe et al., 2007; Benoit, 2008) and the following of rules (Crescentini et al., 2011). While a few of these essentially operational terms do not specifically refer to planning, all describe elementary processes that can be easily identified as necessary components for the formation, maintenance and execution of a plan.

The role of BA10 in planning is further emphasized in the conclusions of many authors. For example, the role of the rPFC has been described as, “essential to support functional connectivity among task-related regions” (Rowe et al., 2007), providing “contingent interposition of two concurrent behavioral plans or mental tasks” (Koechlin and Hyafil, 2007), engaging in “self-generated thoughts” and “spontaneous thoughts” (Dumontheil et al., 2010b), “episodic prospection” and “autobiographical memories” (Benoit et al., 2010), “fluid intelligence” and “prospective imagination” (Roca et al., 2010; Burgess et al., 2011), participating in a “rostrocaudal hierarchy” (Badre and D’Esposito, 2009; Colombo et al., 2015), acting as a “multiple demand” center that monitors the content of cognitive operations, directing mental focus to produce a robust separation of the component sub-goals required for successful completion of a task (Duncan, 2013) and seemingly in deference to the multidomanial complexity of both planning and relative size of BA10, representing “the highest levels of human cognition” (Burgess et al., 2007a). Again, each of these terms and phrases either directly implicate BA10 in planning or they allude to its extraordinary organizational power in orchestrating our behavior around our intentions.

### Temporal Intelligence and the Subdivision of Labor

Since these early studies, many investigations have attempted to detect a subdivision of labor within BA10 that might reveal how this area participates in planning. Using regional cerebral blood flow, Burgess et al. (2003) were able to show that the lateral portion of BA10 became activated in paradigms that required cognitive maintenance (or a prospective memory) of a plan while the superior medial portion of BA10 was either unaffected or showed a decreased level of activity over its resting state (Burgess et al., 2003). These results have since been confirmed and extended in a number of studies (Simons et al., 2006; Rowe et al., 2007; Benoit et al., 2012) which have shown that while activation of the cytoarchitectonically-identifiable, lateral portion of BA10 (lateral rPFC, lrPFC) relates to the subject attempting to keep in mind the requirements for completing a given task, the cytoarchitectonically-identifiable superior medial portion of BA10 (medial rPFC, mrPFC) remains responsive to other, unrelated yet ongoing, environmental information. In what has been called the *Gateway Hypothesis*, Burgess et al. (2007a,b) have proposed what is essentially reciprocal inhibition between the lateral part of BA10 that is responsible maintaining those thoughts related to achieving the current goal (referred to as “internal thought processes”) and the medial part of BA10 that is responsible for directing our attention to external events (Burgess et al., 2007a,b). In terms of survival, such a system would ensure that when we are suddenly under threat from some novel environmental source, our attention would be directed away from the otherwise blind pursuit of our immediate plan.

### The Adaptive Value and Evolution of Temporal Intelligence

In humans, BA10 is larger than any other area of Brodmann. Stereological measurements of the volume of BA10 in a single human hemisphere, showed that it consists of 14.2 mm^3^ of gray matter (Semendeferi et al., 2001). When this volume (14.2 mm^3^) was expressed as a percentage of the total brain volume of the same individual, BA10 of one hemisphere constituted 1.2% of that person’s entire brain. At 2.8 mm^3^, the bonobo has the second largest BA10, constituting 0.75% of its entire brain volume, followed by the chimpanzee (2.3 mm^3^, 0.57%), the gorilla (1.9 mm^3^, 0.54%) and the relatively small-brained Orangutan wherein BA10, although only 1.6 mm^3^, nevertheless, constitutes 2.87% of its brain volume. While in apes and humans BA10 covers the frontal pole of each hemisphere, in the gibbon, a lesser ape, BA10 is confined to the orbital sector of the frontal lobe and markedly reduced in both absolute (0.2 mm^3^) and relative size (0.2% of whole brain volume; Semendeferi et al., 2001). It has long been known that the size of BA9 and BA46 varies radically between different individuals (Rajkowska and Goldman-Rakic, 1995). It is, therefore, highly likely that the size of BA10 would show a similar, high degree of inter-individual variation—a wide spectrum that might well reflect the concomitant variation in the concern with time and planning, shown by different individuals and populations (Lippincott and National Maritime, 1999).

In humans, it has been estimated that BA10 contains around 240,000,000 neurons. However, at approximately 34,000/mm^3^, the density of neurons in BA10 is substantially less than the mean of approximately 52,000/mm^3^ that has been reported for BA46 (Selemon et al., 1995, 1998). Semendeferi et al. (Selemon et al., 1995) have suggested that BA10 is largely composed of neuropile which is indicative of its capacity to make an unusually large number of connections with higher-order association areas—a view supported by the relatively low density of neurons and their abnormally extensive dendritic arborizations (Jacobs et al., 2001; Travis et al., 2005).

In apes, BA10 is many times larger than the area identified as BA10 in new and old world monkeys (Petrides and Pandya, 1999; Semendeferi et al., 2001). While some evidence exists that apes are capable of planning (Mulcahy and Call, 2006), the evidence that monkeys are capable of making plans is controversial (Byrne, 1995; Naqshbandi and Roberts, 2006; Premack, 2007). This may reflect the fact that cytoarchitectonically, the relatively tiny BA10 of the monkey resembles the mrPFC in humans and not the human lrPFC, which has been repeatedly implicated in retaining task specific information (Carmichael and Price, 1994, 1996; Simons et al., 2006; Rowe et al., 2007; Tsujimoto et al., 2010, 2011; Benoit et al., 2012). According to Tsujimoto et al. (2011), neurons in the FPC of the monkey, unlike neurons elsewhere in its PFC, do not show prospective coding (the representation of a place or object as a goal). However, Tsujimoto et al. (2011) go on to propose “…that the FPC [in the monkey] encodes goals generated by certain cognitive processes and that it helps link those processes to particular outcomes… On this view, the FPC promotes future prospective coding elsewhere in the brain without its neurons engaging in ongoing prospective coding” (Tsujimoto et al., 2011, p. 174). According to this theory, even the relatively small volume of PFC constituting BA10 in the monkey has an, albeit limited, capacity to play an integrative rather than an executive role in the temporal organization of behavior. From their studies of BA10 in apes and humans, Semendeferi et al. (2001) conclude that: (a) “During hominid evolution, area 10 underwent a couple of additional changes: one involves a considerable increase in overall size, and the other involves a specific increase in connectivity‥.” and (b) that these changes underwrite the fact that, “Planning of future actions and the undertaking of initiatives are hallmarks of human behavior…” (Semendeferi et al., 2001, p. 240).

### Temporal Intelligence and the Diversity of Planning Across Domains

Planning is essential in all domains of human life, including our endeavors in the social, practical and abstract realms of existence. However, BA10 has been shown to participate in a number of experimental paradigms that neither directly negate nor implicate this area in the orchestration of planning. Thus, neuroimaging studies have shown activations of BA10 when subjects are making simple decisions (Daw et al., 2006), considering moral dilemmas (Raichle and Snyder, 2007), improvising music (Limb and Braun, 2008), making value judgments (Zysset et al., 2002; Lawrence et al., 2009), “mentalizing” (Gilbert et al., 2006; Burgess et al., 2009; Benoit et al., 2010; Roca et al., 2011) and employing and detecting deception (Ganis et al., 2003; Karim et al., 2010).

That humans typically make plans in all domains of existence greatly complicates the interpretation of neuroimaging studies of planning. This difficulty arises because a human subject cannot plan without reference to specific entities, be they feelings, objects or ideas. The human mind has evolved *in situ* and, for this reason, cannot create a plan without making reference to elements of the plan, irrespective of whether they are the psychosocial, practical or abstract realms. This not only complicates the interpretation of neuroimaging studies but it probably explains why studies of planning in children of different ages are typically plagued by issues that appear to be related to the stage of cognitive development of that particular group of children (Friedman and Scholnick, 1997). Each Piagetian stage of cognitive development must surely be accompanied by new procedures of planning that are specifically tailored to the forms of thought that develop in the preoperational (psychosocial), concrete operational (practical) and formal operational (hypothetical) periods (Piaget et al., 1970, 1973). That the rPFC continues to develop throughout childhood and into adolescence with the rate of development peaking in late childhood (Sowell et al., 2004; Konrad et al., 2005; Shaw et al., 2008; Moriguchi and Hiraki, 2011, 2013; Tanaka et al., 2012; Mills et al., 2014) is indicative of the need for the algorithms of planning to be adapted and extended in order to facilitate the sequential maturation of the psychosocial, practical and abstract domains of human intelligence.

The essence of all planning is time. Patients with circumscribed lesions within BA10 of the right rPFC not only showed a deficit in time-based, prospective memory tasks (words and pictures) but also were significantly impaired in the estimation of time (Volle et al., 2011). In contrast, damage to the dorsolateral convexity (BA9/46) produced a failure to manipulate information, but did not disrupt that patient’s ability to estimate time (Ptak and Schnider, 2004). In the physical domain, where the rules of common-sense logic and the essentially Newtonian properties of objects and space are of great importance, chronological time (or objective time) is critical to the efficiency of completing any task. In contrast, within the psychosocial domain, the passage psychological time (subjective time) is far more dependent on our emotions, moods and the company we are keeping (Droit-Volet, 2012). To create an efficient plan in the social realm requires a set of algorithms that incorporate the highly subjective nuances of psychological time (Craig, 2009; van Wassenhove, 2009; Wittmann, 2009, 2013). Finally, when it comes to orchestrating and maintaining the mental focus required to systematically solve abstract problems, BA10 is activated along with BA46 (Wolf et al., 2010). For example, when subjects practiced strategic deception in a two-person bargaining game there was a cooperative activation of BA10 and BA46 (Bhatt et al., 2010). Given that BA10 is always activated in planning paradigms, it seems reasonable to conclude that BA10 is participating in the planning of strategies, the essence of which are being conducted within BA46, an area that is activated when there is demand for executive control (Kübler et al., 2006). The participation of BA46 in tasks that require intense mental concentration suggests that the passage of time during ideational planning would be most likely measured by the level of mental fatigue (Zarahn et al., 2005; Schnell et al., 2007; see: “The Origin of Hypotheses: The Abstract Mind” Section).

People vary vastly in their concern for the passage of time and their propensity to engage in planning (Lippincott and National Maritime, 1999). With less than 1/6 of the volume of gray matter that composes BA10 in humans, apes are only capable of making the simplest plans (Byrne, 1995; Premack, 2007). The massive allocation of cortex to BA10 in the human brain, underscores the absolute dependency of *Homo sapiens* on planning in every domain of existence (Semendeferi et al., 2001), a dependency that can be traced back 28,000 years to the endeavors of Paleolithic Europeans to systematically measure calendric time (Eccles, 1989). As BA10 has been consistently shown to: (a) be necessary for planning; and (b) to be engaged whenever there is a cerebral necessity to temporally organize behavior, I propose that this unique, complex and anatomically-distinct element of human cognition be known as the temporal mind, an organ of the brain that is responsible for our high degree of temporal intelligence (Figure 1).

## The Origin of Hypotheses: The Abstract Mind

In 1781, the German philosopher, Immanuel Kant, published his Critique of Pure Reason wherein he proposed that our complex reasoning stemmed from two elements: an *a priori* or preexisting element and an *a posteriori* or experientially acquired element (Kant et al., 1998). Kant envisaged that this *a priori* element did not arise from our worldly experiences but that it constituted the structural foundation of our capacity to think at the level of ideas and theories. He further postulated that this *a priori* element came into existence to accommodate our inevitable encounter with the intangible phenomena of space and time; asserting that these, essentially invisible properties of the universe are critical to establishing the substrate upon which all complex (hypothetical) reasoning is dependent. By intangible phenomena Kant meant aspects of our environment that, unlike color, size, form, sound, odor, taste and texture, we cannot observe directly but which we believe or hypothesize must exist in order to explain certain aspects of reality. Examples of other intangible phenomena would be gravitational forces, the nature of matter, magnetism, consciousness and thought. As intangible phenomena cannot be perceived, then, as Kant postulated, they can only ever be the hypothetical products of an operational system capable of complex reasoning: a form of reasoning that is fully enabled by end of Piaget’s formal operational phase of cognitive development around approximately the age of 15 years (Piaget et al., 1970, 1973).

### Neurodevelopmental Plasticity and The Conceptualization of Space and Time

It is well known that while the development of cortical cytoarchitecture develops relatively independently of afferent input (Wise and Jones, 1978; Snow and Wilson, 1991; Woo and Finlay, 1996), an essential stage in the postnatal development of the cortex being the reorganization of its neural circuitry in response to the arrival of afferent projections and the receipt of the information these afferents deliver from other neural centers (Snow and Wilson, 1991; Windrem and Finlay, 1991; Li et al., 2013; Vue et al., 2013). Prior to the maturation of the ability to entertain hypothesis, time and space are the only intangible phenomena that can gain entry to the cortex and so influence the post-natal development of the PFC (van Wassenhove, 2009; Wittmann, 2009, 2013). In Piaget’s words, “As Kant has shown so clearly, time and space are not concepts but unique “schemes”, there is only one time and one space in the entire universe” (Piaget and Pomerans, 1969, p. 33). It follows that the identity of the cognitive center responsible for the formation of hypotheses may be revealed by: (a) its maturation around the end of Piaget’s formal operations stage of cognitive development (15 years); and (b) its receipt of powerful afferent inputs that convey information about space and time devoid of perceptually-relevant information about the external environment (Milner and Goodale, 2008). Implicit in the present proposal is the reasonable assumption that, during early postnatal development, inputs to this area of cortex will be accommodated by modifications to its primordial neural circuitry. Much as Kant reasoned long ago, it is difficult to see how else a cortical area capable of engaging in hypothetical thought might be fashioned, other than by being presented, in early development, by the intangible, perceptually-incongruous aspects of space and time.

The critical clue to identifying this area can be found in the organization of the primate visual system (Ungerleider et al., 1998). In their Figure 1, all the perceptual elements that compose human vision are accounted for by the specific visual representations within different post-prefrontal areas. In addition to these, however, there remain two strong anteriorly-directed, visual projections, one to BA46 and the other to BA12 (the homolog of BA47 in humans), prefrontal areas that have been repeatedly implicated in human cognition. These two projections are: (a) the ventral stream, which carries visual and auditory information about the identity of objects and their spatial relationships, constituting the “what” pathway of Goodale et al. (1991), Goodale and Milner (1992) and Milner and Goodale (2008), Petrides and Pandya (1999, 2002; see: “The Origin of Practicality: The Material Mind” Section); and (b) the dorsal stream which carries absolute spatial and temporal information in real-time, constituting the “where” pathway (Goodale et al., 1991). The information from the ventral stream is delivered to BA47/12 (see: The Origin of Practicality), while the information from the dorsal stream is delivered to BA46 (Ungerleider et al., 1998).

In their recent review Milner and Goodale (2008) suggest that the ventral stream provides “vision for perception” while the dorsal stream provides “vision for action”. The critical distinction between these pathways is that, while pivotal in orchestrating behavioral output (movement), information carried by the dorsal stream does not reach awareness and therefore, “is not perceptual in nature” (Milner and Goodale, 2008, p. 775). Indeed in relation to the information being delivered to BA46 along the dorsal stream, they state that “although we may be conscious of the actions we perform, the visual information used [by BA46] to program and control those actions can never be experienced” (Milner and Goodale, 2008, p. 776), citing as supportive evidence Weiskrantz’s assertion that the dorsal stream participates in blind sight (Weiskrantz, 1997, p. 138). As all elements of visual perception, including spatial awareness, are adequately accounted for by the post-prefrontal visual representations (Ungerleider et al., 1998), there remains only one possible role for further integration at the level of the PFC, that of the conceptualization of information—conceptualization of form and function in BA47 (see: “The Origin of Practicality: The Material Mind” Section) and conceptualization of space and time in BA46. Denied information from the ventral stream about physical objects, the primordial BA46 must adapt its circuitry by accommodating the information about time and space from the dorsal stream that, like all intangible phenomena, lacks a perceptual reality (van Wassenhove, 2009). In this process BA46 would necessarily become tuned to the integration (conceptualization) of any and all intangible phenomena into abstract concepts, many of which will be the synchronous, yet dissonant, inputs from the other three cognitive areas (BAs 9, 10 and 47), each relaying integrated (conceptualized) information from a distinctly different domain of life.

In this model, the emergent circuitry within BA46 would be generically adapted to embrace all perceptual and/or cognitive incongruences, such as, for example, the existence of magnetic fields that can only be represented in a unique class of concepts that are, in essence, cognitively-structured hypotheses (Medin et al., 2000). In accordance with Kant’s proposal, the conceptualization of perceptually-barren information about time and space would constitute the vital formative process within BA46, establishing it as the neural center with the capacity to underwrite our ability to entertain any and all hypotheses; an area that begins to dominate behavior around the age of 15, enabling Piaget’s formal operational stage of cognitive development. Of all areas of the cerebral cortex, only BA46 fulfills these critical criteria.

### Neuroanatomy, Development and Evolution of Abstract Intelligence

Walker described area 46 in the monkey PFC as flanking the principal sulcus for almost its entire length (Walker, 1940). In their seminal revision of the cytoarchitecture of the monkey and human PFC, Petrides and Pandya (1999) reported that the caudal moiety of Walker’s area 46 shared some cytoarchitectural characteristics with area 9. Thus, in both monkey and human they define area 46 as cortex flanking the anterior part of the principal sulcus, referring to the cortex flanking the caudal part as area 9/46 (Petrides and Pandya, 1999; Petrides et al., 2012). That areas 46 and 9/46 may share considerable homogeneity in function is indicated by subsequent studies which have shown powerfully interconnects between these abutting areas on both sides of the principal sulcus while both areas 46 and 9/46 share connections to the IPL and to all other areas of Brodmann within the PFC (Yeterian et al., 2012). Unfortunately, some brain imaging studies of these regions have not made specific reference to Brodmann’s areas, opting instead to reference the location of cortical activity to surface structures of the PFC, such as the DLPFC or the middle frontal gyrus. Other brain imaging studies have not attempted to differentiate between areas 46 and 9/46, referring to area 46 of Walker (Walker, 1940) or the DLPFC, as either BA46 (Koch et al., 2006) or BA9/46 (Thakral and Slotnick, 2009; Wolf et al., 2010). Thus, in the following sections, the term BA46-9/46 will be used to refer to area 46, as defined by Walker, a part of the dorsolateral convexity that includes both BA46 and BA9/46, as defined by Petrides and Pandya (1999). It should be cautioned that this is not intended to mean that BA46 and BA9/46 of Petrides and Pandya (1999) will not eventually be shown to have distinct functional properties but that the differences between them are probably not “dominial”, such as those that distinguish the functions of BAs 9, 10 and 47, from each other.

Phylogenetically, the cortex surrounding the principal sulcus is considered to be one of the last areas to evolve, being absent in the primitive primate, the bush baby, Galagos (Preuss and Goldman-Rakic, 1991). Ontogenetically, the cortex surrounding the principal sulcus (BA46-9/46) is one of the last areas of the brain to reach maturity, the structural and functional differentiation of the dorsolateral convexity, including BA46-9/46, remaining incomplete until late puberty (Bonin, 1950; Huttenlocher, 1979; Fuster, 1995; Huttenlocher and Dabholkar, 1997; Giedd et al., 1999; Sowell et al., 1999a,b; Spence et al., 2004; Diamond, 2000; Giedd, 2004; Lenroot et al., 2009; Petanjek et al., 2011; Raznahan et al., 2011). However, this does not mean that these areas are dormant until puberty. Thus, neurodevelopmental studies show that structural and functional changes in the DLPFC are ongoing throughout childhood and are accompanied by the slow but continuous emergence of executive functions that are typically associated with BA46-9/46 (Casey et al., 1997, 2000; Sowell et al., 2001; Adleman et al., 2002; Diamond, 2002; Kwon et al., 2002; Huizinga et al., 2006; Kübler et al., 2006; Chase et al., 2008; Volle et al., 2008; Roca et al., 2010; Rosenberg-Lee et al., 2011; Baron and Leonberger, 2012; Gerbella et al., 2013; Dumontheil, 2014).

### Cognitive Challenges and The Activation of Abstract Intelligence

While it is difficult to demonstrate that monkeys can entertain hypotheses about intangible phenomena, single unit recordings in the DLPFC and/or BA46-9/46 have shown this area to be involved in spatial WM (Levy and Goldman-Rakic, 1999), the generation of behavioral plans (Hoshi and Tanji, 2004), the monitoring of internal representations to guide behavior (Petrides, 2000), executive functions and the higher-level integration of movements (Gerbella et al., 2013), abstract aspects of conceptual processes (Tanji and Hoshi, 2008), a context-dependent, flexible behavioral control system (Tsujimoto and Sawaguchi, 2005) and executive functions and cognitive performance (Opris et al., 2012; Opris and Casanova, 2014). Futhermore, the participation of the monkey PFC in cognitive processes has been further demonstrated by Opris and Casanova (2014) who have shown that microstimulation in the infragranular layers, improves performance on delayed match to sample trials (Opris et al., 2005; Hampson et al., 2012).

In humans, brain imaging has revealed that this area of the PFC is activated in many different experimental paradigms, each revealing a specific role in meeting the cognitive challenge associated with a particular task. Thus, BA46-9/46 has been reported to engage in tasks requiring, strategic deception in a two-person bargaining game (Bhatt et al., 2010), concentration (Koch et al., 2006), sustained monitoring and detection of visuomotor incongruence (Schnell et al., 2007), arithmetic processing (Menon et al., 2002), sustained attention to motion (Thakral and Slotnick, 2009), imaging moving stimuli (Roland and Gulyás, 1995; Goebel et al., 1998; Schendan and Stern, 2007), counteracting susceptibility to proactive interference (Wolf et al., 2010), integration demand and expectation of integration (De Pisapia et al., 2007), high-level cognitive planning and mental manipulation (Amiez and Petrides, 2007), maintenance of WM and concentration requiring mental effort (Zarahn et al., 2005), decisional processes governing oculomotor behavior (Pierrot-Deseilligny et al., 2005), language switching (Wang et al., 2007), maintenance of task performance in Tourette’s syndrome (Marsh et al., 2007), selection from WM and of movements in willed task actions (Rowe et al., 2000), active maintenance of distractor-resistant memory (Sakai et al., 2002) and data-driven scientific discovery (Zhong et al., 2011). Activation of the DLPFC or middle frontal gyrus (often interpreted as homologous to BA46-9/46) has been reported when a task required executive control (Kübler et al., 2006), low confidence, high risk decisions requiring concentration (Fleck et al., 2006), verbal fluency (Abrahams et al., 2003), manipulation of information (Marvel and Desmond, 2010), the mental rotation of objects (Just et al., 2001), automatic retrieval of technical problems and breaking of mental sets (Dandan et al., 2013), self-control (Friese et al., 2013), higher levels of cognitive control/processing (Volle et al., 2008), a domain-independent, extra-mnemonic device to focus attention on items to be remembered (Curtis and D’Esposito, 2003), inhibition of the stereotyped responses (Kadota et al., 2010), higher-order rule switching (Yoshida et al., 2010) and critical cognitive control (Koric et al., 2012). Lesions affecting BA46-9/46 have been reported to compromise performance in simple spatial tasks suggesting deficits in strategy or goal-based dysfunction (Bor et al., 2006), short-term memory and the implementation of algorithmic strategy (Chase et al., 2008), the scheduling of actions based on online manipulation and maintenance of ongoing information (Ptak and Schnider, 2004), higher levels of cognitive control (Volle et al., 2008), executive function and fluid intelligence (Roca et al., 2010) and creative thinking (Colombo et al., 2015).

In general, these studies describe a BA46-9/46 that is activated incrementally by cognitive load, indicating its participation in maintaining mental effort, concentration, WM and attentional focus all in the service of its overall role as the brain’s executive center, upon which rests the responsibility of strategic control over behavior. The activation of BA46-9/46 by, for example, low confidence decision making, switching of language, manipulating information, evaluating equations, focusing of attention or developing a strategy or executive control, again indicate that beyond the need to elaborate the appropriate behavior, this area is little concerned with the concrete details of the material world. Rather, this area appears to be engaged in elements of reason that involve the evaluation and integration of theories, ideas or hypotheses in a quest to formulate what can never be more than a hypothesis regarding, most often, the behavioral strategy most likely to bring reward to its host. One might postulate that within BA46-9/46 entities such as space, form, magnitude and time are manipulated as abstractions that embody their generic properties rather than their concrete manifestations at a particular moment (Medin et al., 2000). In this respect, BA46-9/46 differs radically from the focus upon the concrete properties of people and objects that is the primary concern of BA47 (see: “The Origin of Practicality: The Material Mind” Section).

The word abstract, “existing in thought or as an idea but not having a physical or concrete existence” (Stevenson and Oxford University, 2010), best describes the domain of cognition served by BA46-9/46—a domain that is developmentally driven to embody intangible phenomena into what can only ever be a hypothetical entities, be they physical entities, be they physical entities such as gravity or a set of relationships hidden within a mathematical function. Without such a domain of intelligence, we would be unable to create intelligently-contrived hypotheses that are the foundation for all significant scientific exploration (Zhong et al., 2011). I therefore propose that the cortical area responsible for exclusively elaborating the hypothetical, can best be referred to as the abstract mind, an area of the cortex responsible for our abstract intelligence that is a powerful determinate of success in exploring the mysteries of our universe and ourselves (Figure 1).

## Metacognition and General Intelligence

Tests for general intelligence are designed to test a subject’s power of reason, rather than their ability to recall or recognize facts. When brain imaging is conducted on subjects challenged with questions/tasks that are typical of tests for general intelligence or Spearman’s G-factor, activation of BA46 is more prominent than any other prefrontal areas (Duncan et al., 2000; Gray et al., 2003; Wilke et al., 2003; Gray and Thompson, 2004). Moreover, higher IQ has also been associated with the greater activation of the DLPFC (Menon et al., 2002; Cho et al., 2012; Metcalfe et al., 2013) and a greater thickness of the gray matter of the middle prefrontal gyrus (Wilke et al., 2003; Narr et al., 2007; Choi et al., 2008; Karama et al., 2009; MacLean, 2016). Given that metacognitive ability has often been proposed to be related to IQ (Berger and Reid, 1989; Alexander et al., 1995; Veenman et al., 2004), these studies are highly suggestive that BA46-9/46 is critical to both metacognition and general intelligence and that these fundamental elements of cognition are indeed somewhat interdependent.

Disruption of metacognition has been reported in patients with lesions in right BA46 and BA10 while disrupting function in the DLPFC using transcranial magnetic stimulation decreases metacognitive ability (Rounis et al., 2010). Using brain imaging, Baird et al. (2013) have concluded that, “…an individual’s capacity for accurate introspection…is related to the functional integrity of unique neural networks anchored in the medial and lateral regions of the anterior PFC (aPFC)” (Baird et al., 2013, p. 16657). Finally, Fleming et al. (2012) have demonstrated activation of an area covering the boundary between BA10 and BA46 in the right PFC accompanied by increased functional connectivity to BA46 on the left during metacognition (Fleming and Dolan, 2012).

Alternatively, Frith has proposed that metacognition involves our contemplating the thoughts of others, a task known as mentalizing, suggesting that this “…uniquely human ability …has evolved through its enhancement of collaborative decision-making” (Frith, 2012, p. 2213). As reviewed above (see: “The Origin of Emotional Intelligence: The Social Mind” Section), mentalizing or performing Theory of Mind tasks repeatedly activates BA9, thereby invoking the algorithms of emotional intelligence. However, despite being severely compromised in emotional intelligence (BA9) and, consequently, their mentalizing abilities (Fletcher et al., 1995; Goel et al., 1995; Happe et al., 1996; Ohnishi et al., 2000; Shallice, 2001), intellectually high-functioning adults with ASD, self-reported abnormally high metacognitive abilities, presumably due to the retention in ASD of a functional BA46 and in these cases, a high IQ (Grainger et al., 2014).

Many attempts have been made to expand the concept of metacognition, resulting in terms such as “metacognitive regulation” and “metacognitive knowledge” (Pearsall and Trumble, 2002). However, in its simplest form metacognition is defined as “Awareness and understanding of one’s own thought processes” (Stevenson and Oxford University, 2010). As the four cognitive areas, BAs 9, 10, 46 and 47 are all intimately interconnected (Yeterian et al., 2012), metacognition could theoretically occur when any domain of cognition receives processed information from any other cognitive domain. In this way, any one of these areas could engage in contemplation of the cognitive processes (thoughts) being conducted in any of the other three areas. Nevertheless, available data suggest that metacognition is primarily the concern of the abstract mind (BA46-9/46), perhaps working in concert with the temporal mind (BA10) as a necessary part of strategic planning (see: The Origin of the Future: The Temporal Mind Section) thus enabling it to fulfil its function as the ultimate executor of behavior.

**Figure 2:**
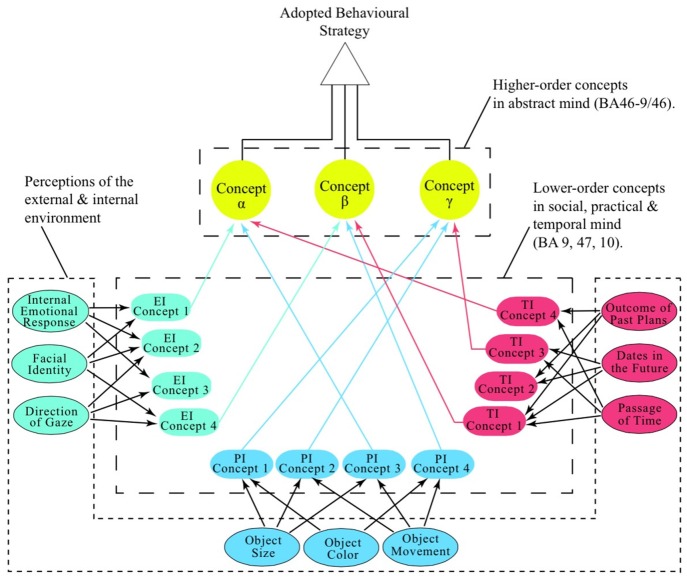
**The construction of abstract intelligence and the origins of metacognition.** Illustration showing how the social, temporal and material domains of cognition (BA9, 10 and 47) form lower-order concepts (see: Social EI, Concepts 1–4; Practical PI Concepts 1–4; Temporal TI, Concepts 1–4) from our perceptions of the world around us. These concepts are lower-order because they are derived from the realities in the psychosocial, temporal and physical realms of existence (ellipses). The abstract mind (BA46-9/46) creates higher-order concepts or “metaconcepts” (α, β and γ) from a synthesis of the conceptual products of the social, temporal and material domains of thought. In this way the abstract mind essentially thinks about the cognitive products of the other three environmentally-linked domains of cognition, thereby creating the almost exclusively human faculty of metacognition.

The findings that BA46-9/46 is primarily involved in metacognition, implies that in its cognitive processes, it reflects upon the cognitively-processed products of the other three cognitive areas, BAs 9, 10 and 47 (Figure 2). It seems that, in everyday life, while the abstract mind is engaged in establishing hypotheses upon which to base future strategies, the social, practical and temporal domains of cognition are receiving and processing information about real, tangible elements within the psychosocial, practical and temporal domains, that otherwise encompass the cognitive life of humans. To put this in the context of world knowledge, BAs 9, 47 and 10 are concerned with establishing lower-order concepts; concepts that are derived directly from, and that therefore embody, aspects of our social, physical and temporal environment, including, in the case of BA9, our internal, emotional reactions to it. In contrast, however, I suggest that the processes in BA46-9/46 are concerned with forming higher-order concepts; concepts that being derived from the integration of sets of lower-order concepts, selected from any or all of the other three cognitive areas (BAs 9, 10 or 47) in circumstances that demand executive action (Figure 2).

It is important to appreciate that when a suite of lower-order concepts arrive at BA46-9/46, they have meaning only in so far as their use in the production of a hypothesis on the best (most rewarding) behavioral strategy, given the prevailing social, practical and temporal circumstances. For example, should Newton’s apple fail to fall to earth, then our material, temporal and, possibly even, our social domains of cognition will be obliged to respond to this novel set of environmental circumstances by embodying them into an entirely novel set of lower-order concepts that will be relayed to the abstract mind. To BA46-9/46, these, temporally-related, lower-order concepts from three radically different domains of cognition (psychosocial, material and temporal) must surely appear as highly incongruent. However, having been developmentally programmed to integrate intangible phenomena, the abstract mind has neural circuitry that has been specifically tailored to create higher-order concepts from dissonant sets of information that, like those arriving from BAs 9, 10 and 47, are many steps removed from the our perceptions of concrete entities such as, color, size, form, sound, odor, taste and texture (see: “The Origin of Hypothesis: The Abstract Mind” Section). In this situation, the abstract mind will create a new higher-order concept that satisfactorily explains why objects do not fall towards the earth—a hypothesis that will obviously be critical to planning future strategies of behavior in this brave new world (Figure 2).

While the circuitries of the neural networks that enable cognition remain a mystery, two very promising lines of investigation are the demonstration that microstimulation within single minicolumns of the monkey dorsolateral convexity facilitates performance on cognitive tasks (Hampson et al., 2012) and the potential to enhance creative thinking in humans by transcranial stimulation of the DLPFC (Colombo et al., 2015). If it can be shown that within BA46-9/46 of humans individual minicolumns are selectively activated by particular hypothetical challenges, we may move one step closer to: (a) understanding the basis of our scientific creativity; and (b) creating an electronic or biological system that, like the human mind, has the capacity to integrate the hypothetical in the production of new testable theories.

## Author Contributions

The author confirms being the sole contributor of this work and approved it for publication.

## Funding

The research for this Hypothesis/Theory Article was funded by the author’s private means. Publication costs will be covered by my institution: School of Medical Science, Griffith University, Gold Coast, Queensland, 4222, Australia.

## Conflict of Interest Statement

The author declares that the research was conducted in the absence of any commercial or financial relationships that could be construed as a potential conflict of interest.
